# Significant Amounts of Functional Collagen Peptides Can Be Incorporated in the Diet While Maintaining Indispensable Amino Acid Balance

**DOI:** 10.3390/nu11051079

**Published:** 2019-05-15

**Authors:** Cristiana Paul, Suzane Leser, Steffen Oesser

**Affiliations:** 1 Independent Nutrition Researcher, Los Angeles, CA 91344, USA; 2 GELITA AG, Uferstrasse 7, 69412 Eberbach, Germany; suzane.leser@gelita.com; 3 CRI, Collagen Research Institute GmbH, Schauenburgerstrasse 116, 24118 Kiel, Germany; steffen.oesser@cri-mail.org

**Keywords:** protein, PDCAAS, protein quality, collagen peptides, functional foods

## Abstract

The results of twenty years of research indicate that the inclusion of collagen peptides in the diet can lead to various improvements in health. According to the current protein quality evaluation method PDCAAS (Protein Digestibility-corrected Amino Acid Score), collagen protein lacks one indispensable amino acid (tryptophan) and is therefore categorized as an incomplete protein source. Collagen protein displays a low indispensable amino acid profile, yet as a functional food, collagen is a source of physiologically active peptides and conditionally indispensable amino acids that have the potential to optimize health and address physiological needs posed by aging and exercise. The objective of this study was to determine the maximum level of dietary collagen peptides that can be incorporated in the Western pattern diet while maintaining its indispensable amino acid balance. Iterative PDCAAS calculations showed that a level as high as 36% of collagen peptides can be used as protein substitution in the daily diet while ensuring indispensable amino acid requirements are met. This study suggests that the effective amounts of functional collagen peptides (2.5 to 15 g per day) observed in the literature are below the maximum level of collagen that may be incorporated in the standard American diet.

## 1. Introduction

Functional foods provide health benefits beyond basic nutrition [1]. The primary role of the diet is to provide sufficient nutrients to meet the nutritional requirements of an individual. However, nutrition science has advanced from the classical concepts of avoiding nutrient deficiencies and basic nutritional adequacy to the concept of optimal health, with the research focus shifting to the identification of biologically active components in foods with potential health benefits or desirable physiological effects [2].

Food-derived bioactive peptides are a product of the hydrolysis of the parent protein source, resulting in specific amino acid sequences that exert positive physiological effects on the body, often distinct from the effects of the individual amino acids they contain. Bioactive peptides are inactive within the native protein, but once cleaved from the native protein by digestion, fermentation, or specific processing, they are shown to produce beneficial effects relating to optimal physical and mental well-being and may also reduce the risk of disease [3,4].

Collagen is a well-established source of functional peptides with biological activity [5]. As functional foods, collagen peptides have been shown to exhibit important physiological functions with a positive impact on health. Numerous studies have shown an improvement in skin elasticity [6], the recovery of lost cartilage tissue [7], reduced activity-related joint pain [8,9], strengthened tendons and ligaments [10,11,12,13], increased lean body mass in elderly men and premenopausal women [14,15], and increased bone mineral density in postmenopausal women [16]. These studies have investigated supplementation with doses of 2.5 to 15 g of bioactive collagen peptides over periods of three to 18 months. The benefits are explained by the ability of bioactive collagen peptides to upregulate the synthesis of extracellular matrix proteins in various tissues via a stimulatory cell effect while providing the specific amino acid building blocks for body collagens [17].

Evidence suggests that the health benefits of collagen peptides support the principle that incorporating such functional components in the daily diet would enhance whole body collagen turnover and other aspects of health more effectively than the current average mix of proteins in common Western diets [18,19,20]. Despite the low indispensable to dispensable amino acid ratio in collagen protein, the Western pattern diet usually contains a significantly high amount of indispensable amino acids, due to high intakes of protein derived from animal food sources [21].

The current method for routinely assessing the adequacy of indispensable amino acids for a given food or diet is PDCAAS (Protein Digestibility-corrected Amino Acid Score), which is due to be replaced by the new approach DIAAS (Digestible Indispensable Amino Acid Score) [22,23]. Despite its limitations [23], PDCAAS has been adopted internationally in food law and policy. In the US regulatory framework, PDCAAS is one of the criteria for identifying and communicating that a food is a “source” of protein for food labeling and marketing purposes [24].

PDCAAS-based protein quality scores are used to adjust dietary protein intakes to meet the daily requirements of indispensable amino acids. Ideally, the amino acid scores (AAS) of a protein or protein mixture should not exceed 1.0, i.e., fulfill 100% of the indispensable amino acid requirements while minimizing excess. This is due to the fact that the body’s metabolic needs include both indispensable and dispensable amino acids [22]. As a consequence, if one or more of the indispensable amino acids are present in excess of requirements, the diet becomes limited in dispensable amino acids, thus unbalanced, even though the PDCAAS remains equal to 1.0 [22]. On the basis of these observations, incorporating functional collagen peptides in the diet without compromising on the indispensable amino acid adequacy can add the nutritional value of dispensable amino acids. 

The objective of this study was to determine the maximum level at which collagen peptides may be incorporated into the typical protein mixture of the standard American diet without lowering the overall PDCAAS score below 1.0.

## 2. Materials and Methods

### 2.1. Composition and Digestibility of the Standard American Diet

The amino acid composition of the standard American diet was obtained from USDA’s (United States Department of Agriculture) 10th nationwide survey, the 1994–1996, 1998 Continuing Survey of Food Intakes by Individuals (CSFII) [25], which was the most recent to report on the average individual amino acid intake.

The digestibility of the standard American diet was set at 96%, as described in the WHO (World Health Organization) Technical Report Series 935 [26] (p. 96).

### 2.2. Composition and Digestibility of Collagen Peptides

The amino acid composition of the collagen peptides was selected from publicly available data on six commonly consumed dietary sources of collagen peptides—four samples from porcine [27], one sample from bovine (GELITA AG, Eberbach, Germany), and one sample from marine [28] origins. The four hydrolysates from porcine collagen were produced using different protease treatments. The sample adopted in this study was that of the collagen peptide that resulted in the lowest proportion of collagen that can be incorporated in the standard American diet while maintaining a high dietary protein quality (PDCAAS equals to 1.0), after iterative PDCAAS calculations were performed for all six samples (Table 1). The collagen peptide selected was sample D from Ao and Li (2012) [27], and its indispensable amino acid composition is presented in Table 2.

The true fecal nitrogen digestibility of collagen was assumed to be at least as high as that of gelatine (98.4%) [29].

### 2.3. Iterative PDCAAS Calculations

Iterative PDCAAS calculations were performed on each of the six collagen peptides according to the guidelines described in the WHO (World Health Organization) Technical Report Series 935 [26] (pp. 94–95). The PDCAAS is the lowest value among all indispensable amino acid scores, corrected by digestibility and truncated to 1.0. The indispensable amino acid scores are obtained by dividing the content of each indispensable amino acid per gram of protein by the corresponding value from the reference amino acid requirement pattern. The reference amino acid requirement pattern used in the calculations was that of children above one year of age and all other older age groups from the DRI (Dietary Reference Intakes) 2005 [30] (p. 689).

The iterative calculations consisted of substituting part of the typical protein mixture of the standard American diet with an arbitrary percentage of collagen peptides as a starting point, such as 10%, and calculating the corresponding PDCAAS, which was equal to 1.0. The collagen peptide percentage was increased by 1% increments as long as the resulting PDCAAS was maintained equal to 1.0. This algorithm identified the maximum amount of collagen peptides that could be incorporated in the diet while maintaining a “high” dietary protein quality [22] (p. 43). In a separate calculation, the percentage of collagen peptides was further increased until the corresponding PDCAAS dropped to 0.75, which identified the maximum amount of collagen peptides that could be incorporated in the diet while maintaining a ”good” dietary protein quality [22] (p. 43).

### 2.4. Collagen Consumption in the Standard American Diet

The average daily collagen protein consumption in the standard American diet was estimated by an analysis of the NHANES (National Health and Nutrition Examination Survey) data from 2001–2002 and 2003–2004 [31,32].

The Recommended Dietary Allowances (RDA) of protein for men (56 g) and women (46 g) aged 19 to 50 years [30] (p. 645) were used to assess whether the effective daily amounts of functional collagen peptides (2.5 to 15 g) observed in the literature were below the maximum level of collagen that may be incorporated in the standard American diet.

## 3. Results

The PDCAAS calculations determined that a level as high as 36% of collagen peptides may be used as protein substitution while maintaining the indispensable amino acid balance and the high protein quality score of the standard American diet (PDCAAS equals to 1.0). The PDCAAS calculation of the daily protein mixture containing 36% collagen peptides and 64% mixed proteins from the standard American diet is shown in Table 2. The first limiting amino acids were the sum of the sulfur-containing amino acids methionine and cysteine. The PDCAAS calculations further revealed that the maximum proportion of collagen peptides that could be incorporated in the standard American diet is 54% while maintaining good dietary protein quality (PDCAAS equals to 0.75). In this case, the first limiting indispensable amino acid was tryptophan for all six collagen peptides (Table 1).

In this study, the individual amino acid scores of the standard American diet ranged from 1.31 to 1.67 (Table 2), indicating an indispensable amino acid surplus of 31% to 67% that allowed for the 36% substitution with collagen peptides, while maintaining the PDCAAS of the diet equal to 1.0.

**Figure 1:**
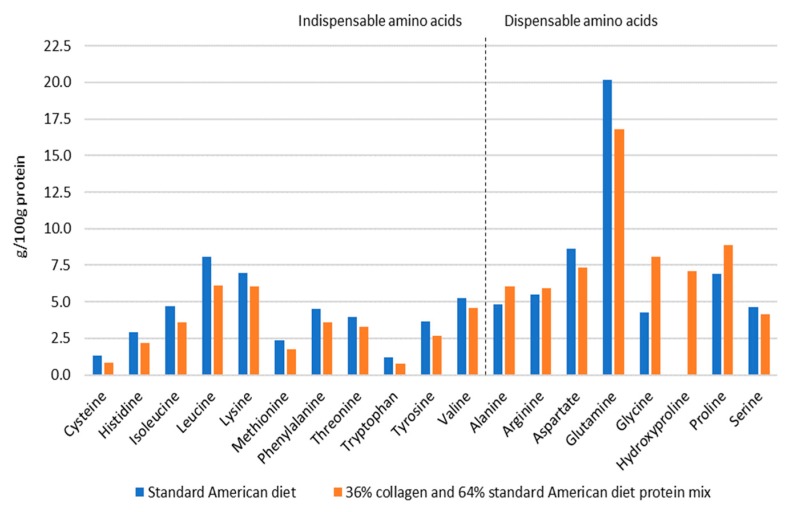
Illustration of the balance between indispensable and dispensable amino acids when the total protein in the standard American diet is replaced with 36% collagen peptides (■) compared to the predominance of indispensable amino acids in the standard American diet (■). Absolute values are based on the amino acid composition of the standard American diet and collagen peptides of porcine origin sample D [27].

Figure 1 illustrates the differences in balance between indispensable and dispensable amino acids, when the total protein in the standard American diet is replaced with 36% collagen peptides. This figure suggests that enriching the diet with effective amounts of collagen peptides could contribute to a better nutritional balance of the twenty dietary amino acids, while maintaining the high protein quality score of the diet.

Table 3 shows the estimated average dietary collagen protein consumption, which varied from 3 g per day for those not consuming sausages or frankfurters in large quantities, to 23 g per day for those consuming these items in significant quantities. This indicates that collagen protein consumption varies widely according to food choices and dietary habits, which may significantly impact on the profile of amino acids obtained from the total protein intake.

When compared to both the minimum RDAs [30] and the actual dietary protein intakes for both men and women in absolute values [33], the effective daily amounts of functional collagen peptides (2.5 to 15 g) observed in the literature [6,7,8,9,10,11,12,13,14,15,16] were found to be below the maximum level of collagen that may be incorporated in the standard American diet (Table 4).

## 4. Discussion

This study addressed the current challenge faced by food manufacturers and healthcare professionals in designing food products and communicating dietary practices for optimal health using functional collagen peptides in compliance with regulatory frameworks that are underpinned by the PDCAAS protein quality evaluation.

The study revealed that including collagen peptides at 36% of total daily protein intake maintains an optimal dietary balance of dispensable and indispensable amino acids (PDCAAS equal to 1.0). Any lower proportion of collagen peptides would maintain the high protein quality of the diet (PDCAAS equal to or higher than 1.0). When taking the amino acid variations in the peptide sequence of collagen peptides into consideration, which are caused by differences in food sources and processing, the estimated range of collagen substitution varied from 36% to 39%, based on the amino acid composition of the six samples of collagen peptides investigated in this study.

Relative to total daily protein intakes, the effective amounts of functional collagen peptides observed in the literature (2.5 g to 15 g) were found to be below the maximum level of collagen that may be incorporated as protein substitution in diets meeting the minimum RDAs for protein [6,7,8,9,10,11,12,13,14,15,16]. In practice, the daily protein consumption in the standard American diet is above the RDA, having increased slightly over the 10-year period from 1999–2008, from 15.6% to 15.9% (100 g) in men, and from 15.2% to 15.5% (67 g) in women, relative to the total energy intake [33]. Recent studies suggest that protein intakes higher than the RDA help promote healthy aging, weight management, and adaptation to exercise [34]. Should the recommended protein intakes increase, the effective amounts of collagen peptides will remain well below the 36% proportion of collagen determined in this study as protein substitution, ensuring that functional collagen peptide supplementation does not pose a problem of overconsumption. On the basis of these observations, effective amounts of functional collagen peptides would be better supplemented rather than substituted in the diet when consuming the RDA levels of protein. This approach would provide all the health benefits associated with collagen peptides while increasing total daily protein intake towards more beneficial levels and improving the dietary amino acid balance.

It is widely accepted that a balance between dispensable and indispensable amino acids is a more favorable metabolic situation than a predominance of indispensable amino acids since indispensable amino acids consumed above the requirements are either converted to dispensable amino acids or directly oxidized [21]. While human physiology includes metabolic pathways for dispensable amino acid synthesis from indispensable amino acids and other precursors, it is still unclear if the body’s proficiency is sufficient to meet the dispensable amino acid needs for optimal health, which may become even more critical with aging, exercise, and disease [18]. Currently, protein quality scores are only determined by the indispensable amino acid content, although the 2013 report on dietary protein quality evaluation in human nutrition from the Food and Agriculture Organization (FAO) recommends that future research is conducted to determine the importance of dietary dispensable amino acid intake, and if there are circumstances in which account should be taken of the dispensable amino acids in calculating the DIAAS of a protein [22]. Also unknown is how the amino acid requirement pattern for optimal health differs from the current basic pattern, requiring more work to improve the general understanding of amino acid needs for different life stages, physiological conditions, and optimal health status [22,35]. New research in this area is needed to provide an up-to-date perspective on protein quality evaluation and categorization that considers the additional health benefits of bioactive peptides [22,36].

The lowest AAS of the standard American diet was estimated at 1.3, indicating a content of indispensable amino acids that is at least 30% above the requirements. Most Western diets have AASs equal to or higher than 1.0 because of high content of animal proteins that contain indispensable amino acids exceeding the requirements [21], and because dietary proteins limited in one amino acid can complement the protein sources that are limited in another amino acid. 

The concern often raised with collagen protein is that a high level of collagen in the diet could lead to a low PDCAAS, mainly because of the complete absence of the indispensable amino acid tryptophan. In theory, PDCAAS equals zero when at least one indispensable amino acid is missing, as is the case with collagen protein. However, as collagen protein is never consumed as the sole or primary source of protein, its nutritional contribution must always be evaluated in the context of a mixed protein diet. As the adult diet is composed of a variety of protein sources, the use in isolation of the PDCAAS value of collagen is of no practical significance. This study showed that even though collagen peptides do not contain tryptophan and are low in cysteine and methionine, the average US diet contains a surplus of these amino acids that allows for the substitution of the total protein intake with 36% to 54% collagen peptides, while maintaining a “good” or “high” dietary protein quality (PDCAAS equals 0.75–1.0). An additional benefit of this substitution may be derived from the increased dietary content of glycine, proline and hydroxyproline, all major components of body collagens, which in turn represent 25–30% of total body proteins [37].

Analysis of NHANES data from 2001–2004 [31,32] revealed that the average collagen consumption varied from 3 g per day for those not consuming significant quantities of sausage and frankfurters, to 23 g per day for those consuming significant quantities of these items, a maximum of 41–50% of the RDA for men and women, respectively, and below the maximum 54% proportion of collagen that can be incorporated in the diet. According to NHANES data from 1999 to 2000 [38], consumption does not seem to have changed in American adults. Other dietary sources of collagen protein include aspic, desserts containing gelatine, or soups with broth from bones or cartilage. However, the collagen in these foods is not hydrolyzed, so they are unlikely to provide reliable concentrations of functional collagen peptides.

## 5. Conclusions

It is beneficial to include functional collagen peptides as part of the daily protein intake, not only for their bioactive properties but also for their rich availability of conditionally indispensable amino acids that may become indispensable under specific physiological situations and life stages. The recommended amount of collagen peptide intake may vary according to the specificity of the peptide (bioactive or non-bioactive), and to the desired health benefit (e.g., skin and nail health, joint health or muscle and bone health). The effective amounts of functional collagen peptides observed in the literature suggest intakes in the range of 2.5 to 15 g daily. These amounts are below the 36% proportion of collagen determined in this study as an adequate substitution in a high-quality protein diet, so that functional collagen peptides may be incorporated in the standard American diet while maintaining indispensable amino acid balance.

## Figures and Tables

**Table 1:** Outcomes from the iterative PDCAAS calculations devised to identify the highest percentage of each type of collagen peptides that may be incorporated in the standard American diet, while maintaining dietary protein quality.

Commonly Consumed Dietary Sources of Collagen Peptides	PDCAAS Equals 1.0 (“High” Dietary Protein Quality)		PDCAAS Equals 0.75 (“Good” Dietary Protein Quality)	
	Collagen (%)	First Limiting Amino Acid	Collagen (%)	First Limiting Amino Acid
Porcine, sample A [27]	39%	Tryptophan	54%	Tryptophan
Porcine, sample B [27]	39%	Tryptophan	54%	Tryptophan
Porcine, sample C [27]	39%	Tryptophan	54%	Tryptophan
Porcine, sample D [27]	36%	Cysteine + methionine	54%	Tryptophan
Bovine (GELITA AG)	39%	Cysteine + methionine	54%	Tryptophan
Marine [28]	39%	Tryptophan	54%	Tryptophan

**Table 2:** The PDCAAS calculation of the daily protein mixture containing 36% collagen peptides and 64% mixed proteins from the standard American diet, based on USDA’s CFSII data from 1994–1996, 1998.

Indispensable Amino Acids	Reference Amino Acid Requirement Pattern * (mg/g)	Standard American Diet Protein Mixture			Collagen Peptides (Porcine Origin, Sample D)			Daily Protein Mixture Containing 36% Collagen Peptides and 64% Standard American Diet Protein Mixture		
		g/100 g	g/100 g Corrected for 96% Digestibility	AAS	g/100 g	g/100 g Corrected for 98.4% Digestibility	AAS	g/100 g	mg/g	AAS
Cys+Met	25	3.68	3.53	1.41	0.72	0.71	0.28	2.50	25.00	1.00 **
Histidine	18	2.91	2.79	1.55	0.85	0.83	0.46	2.08	20.78	1.15
Isoleucine	25	4.70	4.51	1.80	1.61	1.58	0.63	3.44	34.39	1.38
Leucine	55	8.07	7.75	1.41	2.51	2.46	0.45	5.82	58.18	1.06
Lysine	51	6.97	6.69	1.31	4.31	4.22	0.82	5.79	57.92	1.14
Threonine	27	4.00	3.84	1.42	1.96	1.92	0.71	3.14	31.37	1.16
Tryptophan	7	1.20	1.16	1.65	0.00	0.00	0.00	0.73	7.34	1.05
Tyr + Phe	47	8.19	7.86	1.67	2.97	2.91	0.62	6.05	60.55	1.29
Valine	32	5.28	5.07	1.58	3.22	3.16	0.99	4.37	43.70	1.37

Cys + Met = Cysteine and Methionine; Tyr + Phe = Tyrosine and Phenylalanine; AAS = amino acid score; * Reference amino acid requirement pattern (mg/g) from DRI (Dietary Reference Intakes) 2005, for children above one year of age and all other older age groups [30]. ** This AAS represents the calculated PDCAAS of the dietary protein mixture.

**Table 3:** Estimated average daily collagen protein consumption in the standard American diet by males and females, using NHANES data from 2001–2004.

Main Food Groups Sources of Dietary Collagen Protein (NHANES 2001–2004)	Average Collagen Protein (% Dry Weight)	Average Daily Consumption			
		Males		Females	
		Food Group (g)	Collagen Protein (g)	Food Group (g)	Collagen Protein (g)
Beef, pork, veal, lamb, and game	5.15	70.87	3.6	39.69	2.04
Chicken, turkey, and other poultry	1.40	42.52	0.6	34.02	0.48
Seafood	5.50	19.84	1.1	14.17	0.78
Frankfurters, sausages and luncheon meats	55.43	31.18	17.3	17.01	9.43
Total, high consumers of frankfurters, sausages, and luncheon meats			22.6		12.7
Total, no consumers of frankfurters, sausages, and luncheon meats			5.3		3.3

**Table 4:** Effective daily amounts of functional collagen peptides (2.5 to 15 g) observed in the literature, expressed as (A) percent of the Recommended Dietary Allowances (RDA) for both men and women and (B) percent of the average daily protein intake in the standard American diet *.

		Effective Daily Amounts of Functional Collagen Peptides	
		Min 2.5 g	Max 15 g
(A)	RDA (g)	RDA (%)	RDA (%)
Men	56	4	27
Women	46	5	33
(B)	Protein intake * (g)	Protein intake (%)	Protein intake (%)
Men	100	2.5	15
Women	67	4	22

* Daily protein consumption in the standard American diet over the 10-year period from 1999–2008 [33].
